# Comparing Vision-Capable Models, GPT-4 and Gemini, With GPT-3.5 on Taiwan’s Pulmonologist Exam

**DOI:** 10.7759/cureus.67641

**Published:** 2024-08-23

**Authors:** Chih-Hsiung Chen, Kuang-Yu Hsieh, Kuo-En Huang, Hsien-Yung Lai

**Affiliations:** 1 Department of Critical Care Medicine, Mennonite Christian Hospital, Hualien City, TWN; 2 Department of Anesthesiology, DaChien Health Medical System, Miaoli, TWN

**Keywords:** vision feature, pulmonologist exam, gemini, gpt, large language models, artificial intelligence

## Abstract

Introduction

The latest generation of large language models (LLMs) features multimodal capabilities, allowing them to interpret graphics, images, and videos, which are crucial in medical fields. This study investigates the vision capabilities of the next-generation Generative Pre-trained Transformer 4 (GPT-4) and Google’s Gemini.

Methods

To establish a comparative baseline, we used GPT-3.5, a model limited to text processing, and evaluated the performance of both GPT-4 and Gemini on questions from the Taiwan Specialist Board Exams in Pulmonary and Critical Care Medicine. Our dataset included 1,100 questions from 2012 to 2023, with 100 questions per year. Of these, 1,059 were in pure text and 41 were text with images, with the majority in a non-English language and only six in pure English.

Results

For each annual exam consisting of 100 questions from 2013 to 2023, GPT-4 achieved scores of 66, 69, 51, 64, 72, 64, 66, 64, 63, 68, and 67, respectively. Gemini scored 45, 48, 45, 45, 46, 59, 54, 41, 53, 45, and 45, while GPT-3.5 scored 39, 33, 35, 36, 32, 33, 43, 28, 32, 33, and 36.

Conclusions

These results demonstrate that the newer LLMs with vision capabilities significantly outperform the text-only model. When a passing score of 60 was set, GPT-4 passed most exams and approached human performance.

## Introduction

Artificial intelligence (AI) has been widely applied in the field of healthcare over the past years with deep learning, neural networks, and image processing. Notable applications include medical image diagnosis and models predicting mortality rates for specific diseases [1,2]. The emergence of large language models (LLMs) such as OpenAI's Chat Generative Pre-trained Transformer (ChatGPT; OpenAI, San Francisco, CA, United States), which debuted in 2022, has opened up a new field of applications in healthcare.

Accuracy and minimal error margins are paramount in medical diagnosis, making it important to evaluate LLMs’ effectiveness. Some studies have implemented statistical methods such as receiver operating characteristic curves, precision-recall curves, or confusion matrices for assessment. Alternatively, some have assessed LLMs using real medical examination texts. ChatGPT, as the first LLM, was tested with the text of exams for medical staff, primarily focusing on English text. ChatGPT shows a significant improvement in natural language processing (NLP) in 2023, performing at or near the passing threshold for various medical exams without specialized training [3]. It achieves scores equivalent to those of a third-year medical student [4]. In non-English texts, ChatGPT’s performance varies [5]. It has shown proficiency in basic science medical knowledge and applied clinical knowledge [6].

The next generation of language models includes multimodal capabilities, retrieval-augmented generation, and enhanced processing of images, audio, and video. These capabilities are crucial for the medical field, which heavily relies on images and sound. GPT-4 and Gemini, as the next-generation LLMs, offer vision features, handling both text and images. In subspecialties like gynecology, thoracic surgery, radiology, and diagnostic imaging, GPT-4 outperformed GPT-3, but its image processing capabilities are less explored [7-9]. Since Gemini was released in mid-December 2023, there is no related information available yet [10].

For non-English, ChatGPT exhibits lower scores than medical students in the context of simplified Chinese-language medical exams [11]. Chest medicine, gastroenterology, and general medicine are scored relatively well in medical exams in Taiwan. A key limitation is the reliance on non-English text, which may impact performance due to the model’s primary training in English [12]. In subspecialties like family medicine in Taiwan, the results were not satisfactory [13].

In Taiwan’s pulmonary specialist board exam, key areas such as infectious diseases (e.g., pneumonia and tuberculosis), cancer, respiratory disorders, intensive care, sleep medicine, and esophageal diseases are prominent. Building on this study, we focused on non-English texts, specifically chest subspecialties, and utilized next-generation models with vision features. This approach allowed us to incorporate both textual and graphical data into our research.

## Materials and methods

We sourced pulmonary specialist exam questions and answers from 2013 to 2023 from the Taiwan Society of Pulmonary and Critical Care Medicine (TSPCCM) website [14], categorizing them into text- and image-based sections. Two pulmonologists reviewed and subdivided these 1,100 questions into specific topics: infection (bacterial, fungal, and viral origin), tuberculosis (lung and extrapulmonary origin), esophagus topic, thorax anatomy, sleep, pharmacology, lung neoplasms (lung cancer and other origins in thorax), critical care medicine, pathophysiology, mechanical ventilation and oxygen therapy, interstitial lung disease, surgery, pulmonary embolism and vascular disease, asthma, chronic obstructive pulmonary disease (COPD) including bronchiectasis, lung function test, pulmonary vasculitis, autoimmune disease, sarcoidosis and lymphangioleiomyomatosis, pneumothorax and chylothorax, bronchoscopy and image examination, musculoskeletal disease, tracheal disease, pleura disease, diaphragmatic disease, miscellaneous.

We meticulously organized the pulmonary exam questions into a text file, while separately storing the images. Because these one-answer questions have multiple choices to select, the prefix “Please give me only one answer in a single letter form” will be added in the front part of the question content before being fed to LLM. Combine these two parts to form one prompt to input into the model.

**Figure 1:**
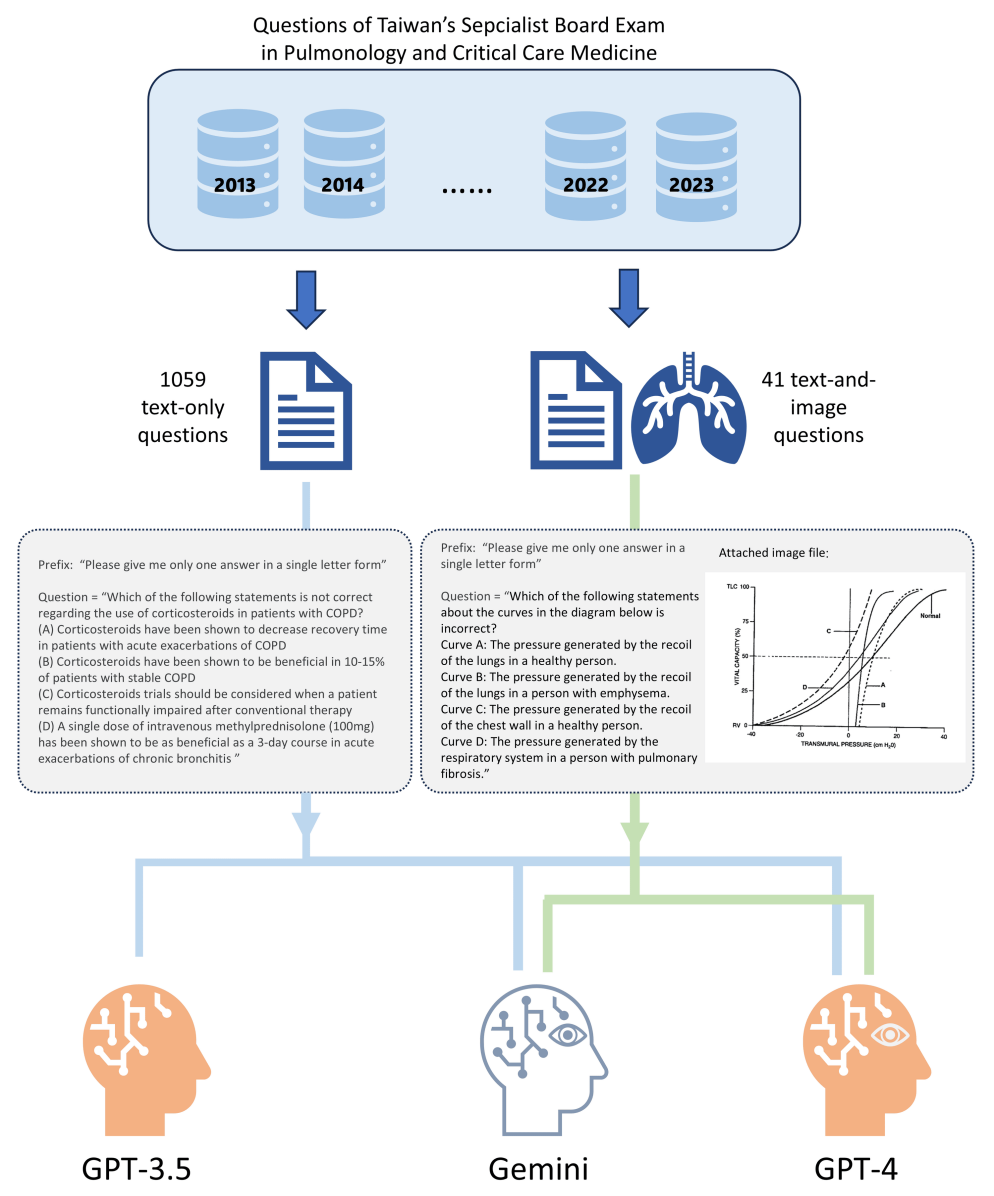
Flowchart of the study

For evaluation, we employed GPT-3.5, which lacks image processing capabilities, alongside GPT-4 Vision and Gemini, both equipped with image functionalities. The text components were analyzed using APIs provided by OpenAI and Gemini. In this study, we used a specific model by setting the model name by API. In ChatGPT, “GPT-3.5-turbo” and “GPT-4” were selected. These two model versions were the same on December 20, 2023. In Gemini, “Gemini-pro” in text-only questions and “Gemini-pro-vision” in questions with both text and images were selected, which was the December 25, 2023 version. For the visual elements in GPT-4, we used the web interface to input text and upload images. The Gemini API, on the other hand, facilitated input for both text and images. The flowchart of the study is presented in Figure 1.

## Results

**Figure 2:**
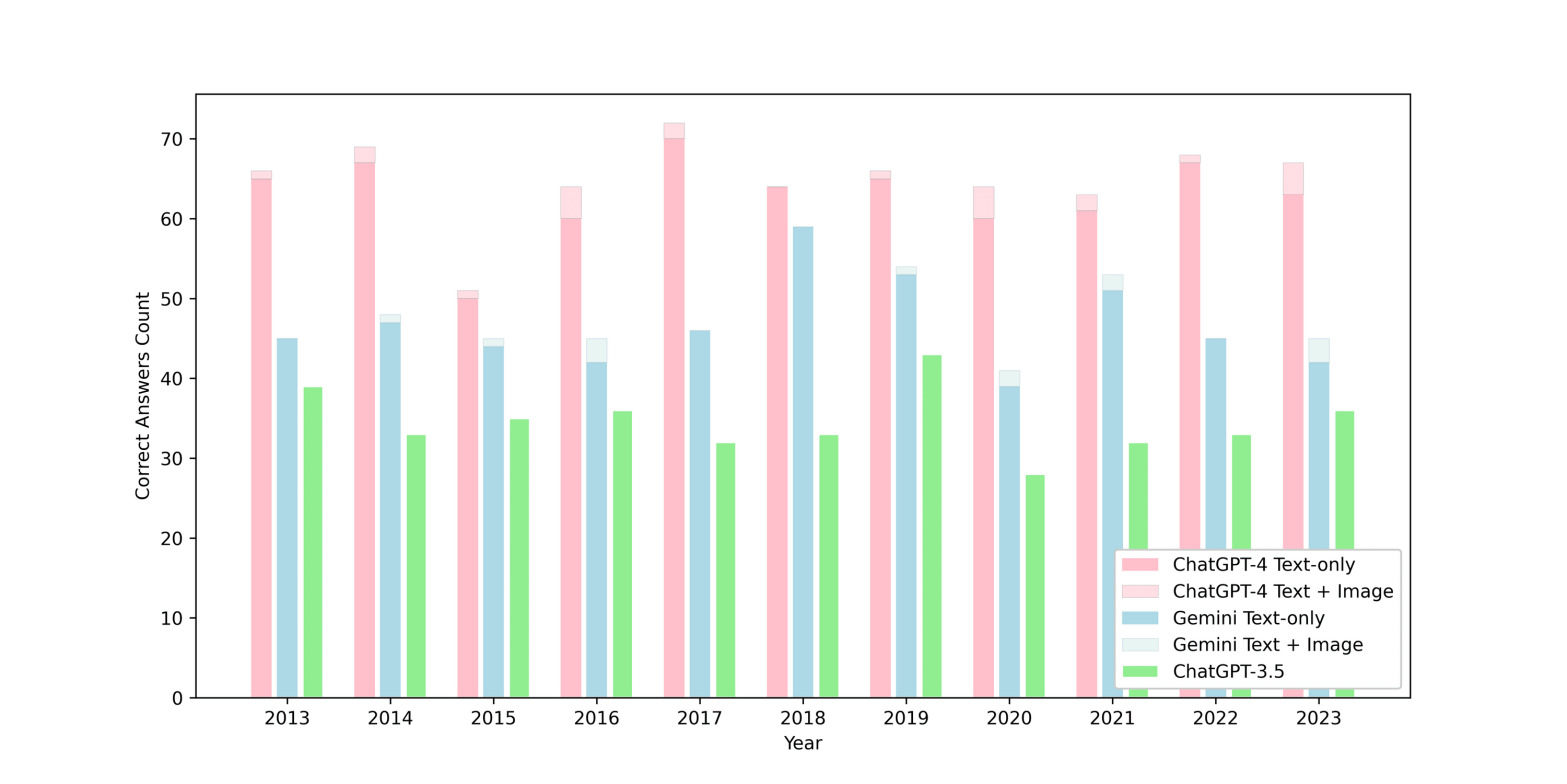
Performance comparison of GPT-4, Gemini, and GPT-3.5 over the years

For each annual exam consisting of 100 questions from 2013 to 2023, GPT-4 correctly answered 66, 69, 51, 64, 72, 64, 66, 64, 63, 68, and 67 questions, respectively, while Gemini scored 45, 48, 45, 45, 46, 59, 54, 41, 53, 45, 45. GPT-3.5 scored 39, 33, 35, 36, 32, 33, 43, 28, 32, 33, 36. The comparison of the three LLMs is illustrated in Figure 2.

For the text-only questions, totaling 1,059 questions, from 2013 to 2023, GPT-4 correctly answered 65, 67, 50, 60, 70, 64, 65, 60, 61, 67, 63, respectively, while Gemini scored 45, 47, 44, 42, 46, 59, 53, 39, 51, 45, 42. For the text and image combined section, totaling 41 questions from 2013 to 2023, GPT-4 correctly answered 1, 2, 1, 4, 2, 0, 1, 4, 2, 1, 4, while Gemini scored 0, 1, 1, 3, 0, 0, 1, 2, 2, 0, 3 (Table 1).

**Table 1:** Scores in text-only and text-and-image questions by year

	2013	2014	2015	2016	2017	2018	2019	2020	2021	2022	2023
Text only											
Total	99	95	96	95	97	99	99	94	96	96	93
GPT-4	65	67	50	60	70	64	65	60	61	67	63
Gemini	45	47	44	42	46	59	53	39	51	45	42
GPT-3.5	39	33	35	36	32	33	43	28	32	33	36
Text and image											
Total	1	5	4	5	3	1	1	6	4	4	7
GPT-4	1	2	1	4	2	0	1	4	2	1	4
Gemini	0	1	1	3	0	0	1	2	2	0	3

In the entire set of exam questions, categorized according to the number of questions, the analysis is as follows: for lung neoplasms (lung cancer and other origins in the thorax), there are a total of 172 questions. GPT-4 answered 124 correctly, Gemini answered 91 correctly, and GPT-3.5 answered 62 correctly. In the section on infections (bacterial, fungal, and viral origin), there are 120 questions, with GPT-4, Gemini, and GPT-3.5 scoring 77, 65, and 35 correct answers, respectively. For critical care medicine, there are 98 questions, with the scores being 66, 45, and 34, respectively. In mechanical ventilation and oxygen therapy, there are 91 questions, with the scores being 60, 39, and 28, respectively. For tuberculosis (lung and extrapulmonary origin), there are 71 questions, with the scores being 38, 32, and 26, respectively. In the topic related to the esophagus, there are 64 questions, with scores of 41, 35, and 23, respectively. For asthma, there are 63 questions, with the scores being 40, 27, and 26, respectively. For COPD, including bronchiectasis, there are 63 questions with scores of 33, 30, and 20, respectively. The details of these results are listed in Table 2.

**Table 2:** Number of correct answers by category COPD: chronic obstructive pulmonary disease

	Total questions	GPT-4	Gemini	GPT-3.5
Lung neoplasm	172	124	91	62
Infection	120	77	65	35
Critical care medicine	98	66	45	34
Mechanical ventilation	91	60	39	28
Tuberculosis	71	38	32	26
Esophageal disease	64	41	35	23
Asthma	63	40	27	26
COPD and bronchiectasis	63	33	30	20
Lung function test	47	28	20	18
Sleep	36	19	13	9
Anatomy	35	23	12	10
Pharmacology	32	21	18	9
Interstitial lung disease	32	22	19	11
Pulmonary embolism and vascular disease	27	21	11	8
Pathophysiology	26	16	13	9
Pneumothorax and chylothorax	23	17	12	10
Surgery	19	12	9	8
Bronchoscopy and image examination	13	8	2	8
Autoimmune disease	12	8	7	3
Sarcoidosis and lymphangioleiomyomatosis	10	9	3	2
Pulmonary vasculitis	8	7	2	3
Pleura disease	8	5	5	3
Trachea disease	2	2	1	0
Musculoskeletal disease	2	1	2	1
Diaphragmatic disease	1	1	1	0
Miscellaneous	25	15	12	14

In the category of questions involving both text and images, the analysis according to the number of questions is as follows: for mechanical ventilation and oxygen therapy, there are a total of 22 questions, with GPT-4 answering 15 correctly and Gemini answering 9. In the sleep category, there are seven questions, with GPT-4 and Gemini scoring 3 and 2, respectively. For other areas, both GPT-4 and Gemini provided correct answers, with details available in Table 3.

**Table 3:** Scores in categories with both text-only and text-with-image questions

	Total question numbers	Image included question numbers	GPT-4	Gemini
Mechanical ventilation	91	22	15	9
Sleep	36	7	3	2
Lung function test	47	4	1	0
Pulmonary embolism and vascular disease	27	3	0	1
Infection	120	1	1	0
Pathophysiology	26	1	1	0
Lung neoplasm	172	1	0	0
Interstitial lung disease	32	1	0	0
Miscellaneous	25	1	1	1

**Figure 3:**
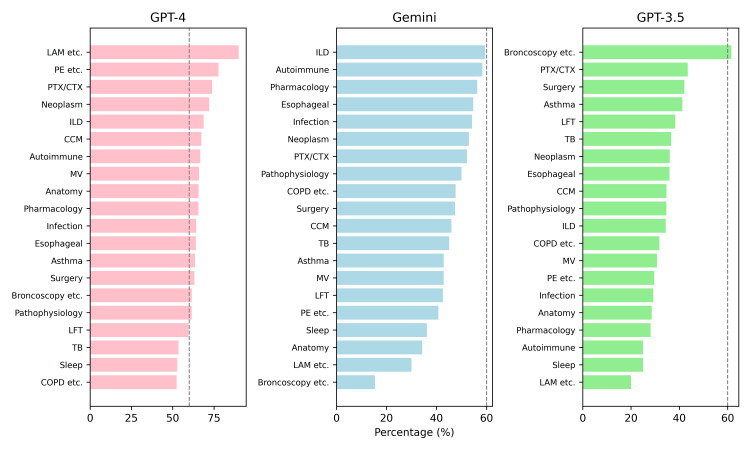
Answer rates in different categories The dashed line represents the 60% passing threshold. Autoimmune: autoimmune disease; bronchoscopy, etc.: bronchoscopy and image examination; CCM: critical care medicine; COPD etc.: chronic obstructive pulmonary disease and bronchiectasis; esophageal: esophageal disease; ILD: interstitial lung disease; LAM etc.: sarcoidosis and lymphangioleiomyomatosis; LFT: lung function test; MV: mechanical ventilation; PE etc.: pulmonary embolism and vascular disease; PTX/CTX: pneumothorax and chylothorax; TB: tuberculosis

We use the total number of questions as the denominator and the number of correct answers as the numerator. Due to the total number of questions being less than 10 and categorized as miscellaneous, we have excluded six categories: pulmonary vasculitis, pleura disease, trachea disease, musculoskeletal disease, diaphragmatic disease, and miscellaneous. Additionally, we have sorted the categories based on the correct answer ratio. With 0.6 as the threshold, the ratios for each category separately for GPT-4, Gemini, and GPT-3.5 are shown in Figure 3. Different preferences for answering questions were observed in these three models.

For categories with more than 60 questions, the answer rates from highest to lowest in GPT-4 are as follows: lung neoplasm, critical care medicine, mechanical ventilation, infection, esophageal disease, asthma, tuberculosis, and COPD and bronchiectasis. In Gemini, the order is esophageal disease, infection, lung neoplasm, COPD and bronchiectasis, critical care medicine, tuberculosis, mechanical ventilation, and asthma. For GPT-3.5, it is asthma, tuberculosis, lung neoplasm, esophageal disease, critical care medicine, COPD and bronchiectasis, mechanical ventilation, and infection (Figure 3).

## Discussion

AI capable of understanding human language has been a focus of research for many decades. Due to the complexity of human language, significant progress in this field remained elusive until the development of the ChatGPT, built on the GPT-3.5 architecture [15]. It has been trained with extensive and massive text data from the internet, making it able to comprehend and respond to human language with remarkable accuracy and efficiency [16].

GPT-4 vision ability represents a significant evolution in language models. Traditionally, such models were constrained to text-based inputs, limiting their application scope. GPT-4 incorporates image processing, thereby enhancing the model’s utility and applicability across diverse scenarios that require multimodal understanding [17]. Gemini, an advanced AI model proposed by Google DeepMind, was introduced on December 6, 2023. It is designed for multimodality, with text, images, videos, audio, and code processing. It also stands out as the first model to surpass human experts in massive multitask language understanding, a key benchmark for AI knowledge and problem-solving [10].

In this study, we categorized the exam questions by topic and observed that the accuracy rates of the three models varied across different subjects. However, for common thoracic conditions such as neoplasms, infections, critical care medicine, and asthma, the accuracy rates were above average, likely due to the abundance of data available for these conditions. Notably, despite the growing evidence linking sleep to various internal medicine conditions, the accuracy rates for this topic were consistently low across all three models. We speculate that this may be due to the general public’s lack of awareness of the importance of sleep medicine, leading to insufficient training data provided by the companies. When all available LLMs have insufficient knowledge on a particular topic, there is a risk of misleading the public. This phenomenon warrants further investigation.

The examination questions are distributed over 11 years, from 2013 to 2023; GPT-3.5, GPT-4, and Gemini show no differences in answer trends over varying years. For categories with more than 60 questions, the answer rates also vary in these three LLMs. This may be due to the differences in the training datasets. A professional medical team preparing relevant data for training could be a direction for future medical LLMs.

Interestingly, the next generation of LLMs, in addition to being multimodal with multimedia input and recognition, also incorporates retrieval-augmented generation (RAG). RAG is an NLP framework that combines search retrieval and generative capabilities [18]. Through this architecture, models can search for relevant information fetched from external databases and use this information to generate responses or complete specific NLP tasks, therefore enhancing the accuracy and reliability of generative AI models [19].

To understand whether the above answers were generated by the original model training due to its knowledge base being up to date only until January 2022, we inputted “102-year chest and critical care medicine specialist physician examination questions” in Chinese words into the ChatGPT web user interface. GPT-3.5 responded that it could not answer questions about the exam of year 102 (the year 102 in the Taiwan calendar, which corresponds to the year 2013 in the Gregorian calendar). GPT-4 was able to search for information through the integrated Microsoft Bing search engine. It successfully found the exam questions and answers files (in PDF file type) on the TSPCCM website. It responded to the correct file links but subsequently failed to find them in later searches again, indicating possible inconsistencies in search results. Gemini exhibited what is known as the “language model illusion problem,” responding with content unrelated to the query [20].

Although GPT-4 and Gemini both possess basic RAG capabilities, they have not yet demonstrated the ability to search medical websites for relevant exam questions and directly analyze corresponding answers. However, we believe that similar capabilities are highly likely to appear in the next generation of language models, making it challenging to assess whether a language model has comprehensive knowledge capabilities. Medical models require highly accurate data and responses from professionals. Due to integration with search engines, the RAG capabilities might introduce problematic information from the internet, leading to issues with answer accuracy.

Over the last year, language models have evolved remarkably due to advancements made by various companies, now featuring capabilities for both text- and image-based analysis. Language models specialized in medical research outperform general models in the medical domain. For instance, Google’s Med-PaLM has already demonstrated this superiority [21]. However, the latest version, Med-PaLM 2, shows even more significant progress in the US Medical Licensing Exam, scoring 86.2 as opposed to Med-PaLM’s 67.2, nearly reaching the expert level [22]. According to our study, there is a possibility that current LLMs may have made notable progress in highly specialized areas and non-English domains. Not only GPT-4, but Gemini, upon its release, had already surpassed GPT-3.5. This could be due to Gemini being the successor model to Med-PaLM.

Limitations

The pulmonary specialist board exam in Taiwan consists of two stages. The first stage includes a written exam, like the questions in this study, and a chest X-ray interpretation test. The second stage is a professional knowledge interview. Although passing the written exam does not guarantee passing the specialist board exam, an important consideration is how to prevent these models from being used in cheating due to their capability to pass written tests. This study does not address the misuse of AI, but it is a challenge that is expected to arise in the future.

## Conclusions

Integrating image processing into these models will enhance their ability to assist healthcare professionals in addressing complex clinical challenges in the future. Our study demonstrates that GPT-4 with vision and Gemini, in both text-only and text-plus-image tasks, outperforms GPT-3.5, which was limited to text-based tasks. As knowledge and training continue to accumulate, future LLMs are likely to excel in both text and medical imaging tasks, indicating a promising future for language models in applications such as medical examinations.
