# Correction: Comparing Vision-Capable Models, GPT-4 and Gemini, With GPT-3.5 on Taiwan’s Pulmonologist Exam

**DOI:** 10.7759/cureus.c191

**Published:** 2024-08-28

**Authors:** Chih-Hsiung Chen, Kuang-Yu Hsieh, Kuo-En Huang, Hsien-Yung Lai

**Affiliations:** 1 Department of Critical Care Medicine, Mennonite Christian Hospital, Hualien City, TWN; 2 Department of Anesthesiology, DaChien Health Medical System, Miaoli, TWN

This article has been corrected at the request of the submitting author to change the affiliation of Hsien-Yung Lai as follows:

Original: Department of Education and Research, Mennonite Christian Hospital, Hualien City, TWN Corrected: Department of Anesthesiology, DaChien Health Medical System, Miaoli, TWN

The authors deeply regret that this error was not identified and addressed prior to publication.

 

